# Supplementary material for: SqueezeCall: nanopore basecalling using a Squeezeformer network
Source: GigaByte. 2025 Feb 14;2025:gigabyte148. doi: 10.46471/gigabyte.148 (PMC11851125; doi:10.46471/gigabyte.148)
Supplement: Peer review history

# Reviewer report: Review MS


Reviewer name and names of any other individual's who aided in reviewerTao JiangDo you understand and agree to our policy of having open and named reviews, and having your review included with the published manuscript. (If no, please inform the editor that you cannot review this manuscript.)YesIs the language of sufficient quality?YesPlease add additional comments on language quality to clarify if neededIs there a clear statement of need explaining what problems the software is designed to solve and who the target audience is? YesAdditional CommentsIs the source code available, and has an appropriate Open Source Initiative license <a href="https://opensource.org/licenses" target="_blank">(https://opensource.org/licenses)</a> been assigned to the code?YesAdditional CommentsAs Open Source Software are there guidelines on how to contribute, report issues or seek support on the code?YesAdditional CommentsIs the code executable?YesAdditional CommentsIs installation/deployment sufficiently outlined in the paper and documentation, and does it proceed as outlined?YesAdditional CommentsIs the documentation provided clear and user friendly?YesAdditional CommentsIs there enough clear information in the documentation to install, run and test this tool, including information on where to seek help if required?YesAdditional CommentsIs there a clearly-stated list of dependencies, and is the core functionality of the software documented to a satisfactory level?YesAdditional CommentsHave any claims of performance been sufficiently tested and compared to other commonly-used packages? YesAdditional CommentsIs test data available, either included with the submission or openly available via cited third party sources (e.g. accession numbers, data DOIs)?Additional CommentsAre there (ideally real world) examples demonstrating use of the software? YesAdditional CommentsIs automated testing used or are there manual steps described so that the functionality of the software can be verified?Additional CommentsAny Additional Overall Comments to the AuthorIn this study, Zhongxu ZHU presents a novel approach combining the Squeezeformer architecture with masking techniques for nanopore basecalling, demonstrating meaningful improvements over existing methods. However, several concerns need to be addressed before publication. 1. The rationale behind the chosen hyperparameter values (e.g., mask_time_prob = 0.05 and mask_time_length = 5) is unclear. Did the authors experiment with other hyperparameter settings? If so, please provide results or justification for selecting these specific values. 2. The signal preprocessing methodology would benefit from a more detailed explanation. Specifically, the current description should clarify whether standard signal normalization techniques were applied to the raw current signals and detail any FFT preprocessing steps. Since nanopore sequencing signals can vary significantly between different species and experimental runs, explaining how SqueezeCall handles these variations would help other researchers implement and potentially improve upon this work. The author could consider including a flowchart or detailed pseudocode of the preprocessing pipeline. 3. A more detailed analysis of the model's error handling would strengthen the paper. Specifically, how effectively does SqueezeCall address key challenges in nanopore sequencing, such as homopolymer errors? 4. The manuscript requires attention to detail in presentation,such as: I) In Table 1, the mismatch rate (3.68) for the NA12878 Human Dataset is partially bolded, which should be corrected for consistency. II) On page 12, line 19, there is an unnecessary "e.g." before "SqueezeCall," which should be removed. 5. Instances of "Error! Reference source not found" are present in the manuscript. Please resolve these citation errors to ensure clarity and credibility.RecommendationMajor Revisions


# Reviewer report: Review MS


Reviewer name and names of any other individual's who aided in reviewerXimei LuoDo you understand and agree to our policy of having open and named reviews, and having your review included with the published manuscript. (If no, please inform the editor that you cannot review this manuscript.)YesIs the language of sufficient quality?YesPlease add additional comments on language quality to clarify if neededIs there a clear statement of need explaining what problems the software is designed to solve and who the target audience is? YesAdditional CommentsIs the source code available, and has an appropriate Open Source Initiative license <a href="https://opensource.org/licenses" target="_blank">(https://opensource.org/licenses)</a> been assigned to the code?YesAdditional CommentsAs Open Source Software are there guidelines on how to contribute, report issues or seek support on the code?YesAdditional CommentsIs the code executable?YesAdditional CommentsIs installation/deployment sufficiently outlined in the paper and documentation, and does it proceed as outlined?YesAdditional CommentsIs the documentation provided clear and user friendly?YesAdditional CommentsIs there enough clear information in the documentation to install, run and test this tool, including information on where to seek help if required?Additional CommentsIs there a clearly-stated list of dependencies, and is the core functionality of the software documented to a satisfactory level?YesAdditional CommentsHave any claims of performance been sufficiently tested and compared to other commonly-used packages? YesAdditional CommentsIs test data available, either included with the submission or openly available via cited third party sources (e.g. accession numbers, data DOIs)?Additional CommentsAre there (ideally real world) examples demonstrating use of the software? YesAdditional CommentsIs automated testing used or are there manual steps described so that the functionality of the software can be verified?Additional CommentsAny Additional Overall Comments to the AuthorThis manuscript describes a tool called SqueezeCall, designed for accurate nanopore basecalling. The authors compare SqueezeCall with four existing basecalling methods across 11 different datasets and report that it outperforms them in terms of basecalling accuracy. However, the study has several shortcomings and requires further clarification. Below are my comments. 1) The current discussion and conclusion section lacks sufficient analysis of the scientific and practical value of the proposed algorithm for nanopore sequencing. To strengthen the manuscript, consider expanding the conclusion section to provide a detailed discussion on the practical applications of the tool in real-world nanopore sequencing workflows. Additionally, include potential directions for further improvement of the algorithm to inspire future research and development in this area. 2) The figures in the manuscript are blurry and should be improved for clarity. Additionally, the layout requires better structuring and alignment, ensuring that the borders are neat and consistent. Efforts should be made to enhance the visual appeal of the figures, and the accompanying descriptions should provide sufficient detail to enable readers to understand the content by reviewing the figures alone. 3)To enhance the showcasing of SqueezeCall's superiority, it is advisable to include one or two of the latest methods for comparison. Minor comments: 1) There are instances of missing punctuation marks in sentences throughout the article. For example, the sentence on page 3, line 9, is missing a period at the end. 2) Address the "Reference not found" issues that appear in several places in the manuscript. 3) Number all formulas in the manuscript for easier reference and citation. 4) Verify that all references are complete and formatted according to the target journal's guidelines. 5) Some areas in Table 1 that necessitate emphasis through bold formatting are inaccurately labeled. 6) Certain content in Figure 1 and Figure 2 appears redundant; consolidation is recommended to streamline the visuals.RecommendationMinor Revisions


# Reviewer report: Review MS


Reviewer name and names of any other individual's who aided in reviewerYongtian WangDo you understand and agree to our policy of having open and named reviews, and having your review included with the published manuscript. (If no, please inform the editor that you cannot review this manuscript.)YesIs the language of sufficient quality?YesPlease add additional comments on language quality to clarify if neededIs there a clear statement of need explaining what problems the software is designed to solve and who the target audience is? YesAdditional CommentsIs the source code available, and has an appropriate Open Source Initiative license <a href="https://opensource.org/licenses" target="_blank">(https://opensource.org/licenses)</a> been assigned to the code?YesAdditional CommentsAs Open Source Software are there guidelines on how to contribute, report issues or seek support on the code?YesAdditional CommentsIs the code executable?YesAdditional CommentsIs installation/deployment sufficiently outlined in the paper and documentation, and does it proceed as outlined?YesAdditional CommentsIs the documentation provided clear and user friendly?YesAdditional CommentsIs there enough clear information in the documentation to install, run and test this tool, including information on where to seek help if required?Additional CommentsIs there a clearly-stated list of dependencies, and is the core functionality of the software documented to a satisfactory level?YesAdditional CommentsHave any claims of performance been sufficiently tested and compared to other commonly-used packages? YesAdditional CommentsIs test data available, either included with the submission or openly available via cited third party sources (e.g. accession numbers, data DOIs)?Additional CommentsAre there (ideally real world) examples demonstrating use of the software? YesAdditional CommentsIs automated testing used or are there manual steps described so that the functionality of the software can be verified?Additional CommentsAny Additional Overall Comments to the AuthorThe manuscript presents SqueezeCall, an innovative approach that combines Squeezeformer architecture with masking techniques for nanopore basecalling. The work demonstrates promising accuracy improvements through comprehensive evaluation across multiple datasets, including human, lambda phage, and nine bacterial datasets. The architecture thoughtfully integrates convolution layers for signal downsampling, employs a Squeezeformer network for capturing global context, and introduces a novel masking technique inspired by Wav2vec2.0. While the research direction and initial results are valuable, several aspects could be strengthened to enhance the work's impact: 1. Several formatting inconsistencies in the manuscript require attention for improved clarity. In Table 1, the mismatch rate (3.68) for the NA12878 Human Dataset is partially bolded, which affects the table's readability. On page 12, line 19, the redundant "e.g." before "squeezecall" should be removed. The citation system needs review as multiple instances of "Error! Reference source not found" appear throughout. 2. The mask hyperparameter selection (mask_time_prob = 0.05 and mask_time_length = 5) requires empirical justification. Including ablation studies showing model performance with different masking probabilities (e.g., 0.01, 0.03, 0.07, 0.1) and lengths (e.g., 3, 7, 10) would provide valuable insights. This analysis could reveal whether the chosen values are optimal or if there's room for improvement. A visualization of how different masking parameters affect model performance could be particularly instructive. 3. The error analysis could be expanded to provide deeper technical insights. The author should particularly analyze the distribution of skip and stay errors in homopolymer regions (e.g., AAAAA or GGGGG) where nanopore basecalling typically struggles. 4. The manuscript would benefit from exploring modified base calling capabilities. The author could train and evaluate the model on datasets containing known DNA modifications (e.g., 5mC, 6mA). This could start with synthetic sequences containing known modifications and extend to well-characterized genomic regions. Even if full modified base calling is beyond the current scope, preliminary results or architectural considerations for future extension would be valuable.RecommendationMinor Revisions


# Reviewer report: Re-Review MS


Indicate in the comments box below whether you are happy with the changes made or if the manuscript is unacceptable.Comments on revised manuscriptThe revised manuscript addresses most of my concerns; however, I have a few additional suggestions before recommending it for publication: 1) The newly added experimental Mask module presents only the results. Charts should be included to provide a more intuitive and visual representation of these results. 2) The images included in the Response should also be incorporated into the main text or published as supplementary materials alongside the manuscript. 3) The formulas in the manuscript are missing corresponding numbers. It is recommended to add numbers to each formula for clarity and ease of reference.


# Editor report: Accept


Editor’s AssessmentThe accuracy of basecalling of nanopore sequencing still needs to be improved. With recent advances in deep learning this paper introduces SqueezeCall, a novel end-to-end tool for accurate basecalling. This uses Squeezeformer-achitecture which integrates local context extraction through convolutional layers and long-range dependency modeling via global context acquisition. Testing and peer review demonstrated that SqueezeCall outperformed traditional RNN and Transformer-based basecallers across multiple datasets, indicating its potential to refine genomic assembly and facilitate direct detection of modified bases in future genomic analytics. Future work is ongoing that will focus on training on highly curated datasets, including known modifications, to further increase research value. SqueezeCall is MIT licensed and available from GitHub here: https://github.com/labcbb/SqueezeCallEditor’s AssessmentThe accuracy of basecalling of nanopore sequencing still needs to be improved. With recent advances in deep learning this paper introduces SqueezeCall, a novel end-to-end tool for accurate basecalling. This uses Squeezeformer-achitecture which integrates local context extraction through convolutional layers and long-range dependency modeling via global context acquisition. Testing and peer review demonstrated that SqueezeCall outperformed traditional RNN and Transformer-based basecallers across multiple datasets, indicating its potential to refine genomic assembly and facilitate direct detection of modified bases in future genomic analytics. Future work is ongoing that will focus on training on highly curated datasets, including known modifications, to further increase research value. SqueezeCall is MIT licensed and available from GitHub here: https://github.com/labcbb/SqueezeCall
